# Whole-Genome Sequences of 15 Strains of *Staphylococcus aureus* subsp. *aureus* Isolated from Foodstuff and Human Clinical Samples

**DOI:** 10.1128/genomeA.00684-15

**Published:** 2015-06-25

**Authors:** Julien Crovadore, Gautier Calmin, Jenna Tonacini, Romain Chablais, Andreas Baumgartner, Bruno Schnyder, Elisabeth Hodille, François Lefort

**Affiliations:** a Plants and Pathogens Group, Research Institute Earth Nature and Environment, hepia, HES-SO University of Applied Sciences and Arts Western Switzerland, Jussy, Geneva, Switzerland; b Faculty of Engineering and Architecture, HES-SO University of Applied Sciences and Arts Western Switzerland, Delémont, Switzerland; c Institute of Life Technologies, hevs, HES-SO University of Applied Sciences and Arts Western Switzerland, Sion, Switzerland; d Group Biology and Biotechnology, Risk Analysis Section, Federal Food Safety and Veterinary Office, Federal Department of Home Affairs, Bern, Switzerland; e INSERM U1111, University of Lyon 1, Lyon, France; f National Reference Center for *Staphylococcus*, Hospices Civils de Lyon, Bron, France

## Abstract

The whole-genome sequences of 15 strains of *Staphylococcus aureus* (10 strains isolated from foodstuff samples in Switzerland and five from human clinical samples) were obtained by Illumina sequencing. Most strains fit within the known diversity for the species, but one (SA-120) possessed a higher G+C content and a higher number of genes than usual.

## GENOME ANNOUNCEMENT

*Staphylococcus aureus* subsp. *aureus*, a pathogenic bacterium, is the cause of a broad spectrum of human diseases ranging from mild skin infections to endocarditis, sepsis, and pneumonia (1), and its pathogenic potential is enhanced by various antibiotic resistances (2). It also causes infections in farm animals, which may be passed to farmers (3), and the contamination of primary food products represents a risk of major concern for public health (4). We report here the sequencing of 10 strains collected from various ready-to-eat food by the Federal Food Safety and Veterinary Office (Switzerland), along with 5 reference strains provided by the French National Reference Center for *Staphylococcus* (France).

Genomic DNA of the 15 *S. aureus* strains was extracted from pure cultures grown to stationary phase, according to an adapted protocol (5). Libraries were constructed using the Nextera XT kit (Illumina, USA). All genomes were sequenced within one Illumina MiSeq run at 2 × 250-bp read length, using a MiSeq reagent kit version 2 (Illumina). Trimming and quality control of the raw reads were performed with FastQC (6). Genome assemblies were computed with the SPAdes genome assembler 3.1 (7). The resulting contigs were arranged with BioEdit (8) and analyzed with QUAST (9). The genomes were screened *in silico* with PlasmidFinder (10) to identify all circular or integrated plasmid genomes. Automated gene annotation was carried out by the NCBI Prokaryotic Genome Automatic Annotation Pipeline (PGAAP) (11) and reviewed with RAST version 2.0 (12).

Mass parallel sequencing produced between 775,000 and 1,785,000 reads of 250 bp for a genome coverage ranging between 69-fold and 140-fold. The total genome lengths ranged between 2,740,099 bp (FRI151m) and 3,012,213 bp (SA-120), which is congruent with the known diversity within this species. Genomes varied between 27 and 174 contigs >500 bp. The G+C content was in the known range for the 38 genome groups of *S. aureus*, except for 3 strains with G+C contents of 33.15% (SA-067 and SA-083) and 35.85% (SA-120). Eight strains (A900624, DSM 799, FRI1151m, SA-022, SA-038, SA-047, SA-067, and SA-260) harbored a single plasmid, while one contained two plasmids (CCM5757). The plasmid lengths varied between 4,566 bp and 37,692 bp (both plasmids from CCM5757). Fourteen genomes contained between 2,823 (FRI1151m) and 3,113 (CCM5757) genes, corresponding to 2,673 (SA-006) to 2,996 (CCM5757) proteins, fitting in the known range for *S. aureus*, while strain SA-120 displayed 3,351 genes for 3,161 proteins, which is outside the common range. None of the strains was methicillin or vancomycin resistant, six strains were resistant to penicillin, and all contained resistance genes for trimethoprim, sulfonamide, quinolone, rifamycin, kirromycin, teicoplanin, and a multiple peptide resistance factor gene. All strains harbored a variety of enterotoxin, exotoxin, and other virulence factor genes, while 2 strains (SA-006 and SA-260) owned a gene for a toxic shock syndrome toxin.

### Nucleotide sequence accession numbers.

The nucleotide sequence accession numbers are shown in Table 1.

**Table 1:** Accession numbers for the strains in this study

Strain name	Genome accession no.	Contig or plasmid accession numbers
A900624	JXHT00000000	JXHT01000001 to JXHT01000082
CCM5757	JXHU00000000	JXHU01000001 to JXHU01000163
DSM 799	JXHV00000000	JXHV01000001 to JXHV01000095
FRI1151m	JXHW00000000	JXHW01000001 to JXHW01000081
FRI137	JXHX00000000	JXHX01000001 to JXHX01000074
SA-006	JXHY00000000	JXHY01000001 to JXHY01000080
SA-022	JXHZ00000000	JXHZ01000001 to JXHZ01000075
SA-038	JXIA00000000	JXIA01000001 to JXIA01000091
SA-047	JXIC00000000	JXIC01000001 to JXIC01000089
SA-067	JXID00000000	JXID01000001 to JXID01000203
SA-083	JXIE00000000	JXIE01000001 to JXIE01000098
SA-085	JXIF00000000	JXIF01000001 to JXIF01000196
SA-120	JXIG00000000	JXIG01000001 to JXIG01000630
SA-210	JXIH00000000	JXIH01000001 to JXIH01000114
SA-260	JXIB00000000	JXIB01000001 to JXIB01000142
